# Confronting food insecurity through agricultural interventions: the Farmer FIRST program in India

**DOI:** 10.3389/fnut.2024.1423599

**Published:** 2025-01-14

**Authors:** Purushothaman Venkatesan, Nilakandan Sivaramane, Ch Srinivasa Rao, Ramanujam Venkattakumar, Sethuraman Sivakumar, Palanisamy Mooventhan, Rajarshi Roy Burman, Bommu Kalyani, Lalitha Navya Challa

**Affiliations:** ^1^ICAR- National Academy of Agricultural Research Management, Hyderabad, India; ^2^ICAR- Central Tuber Crops Research Institute, Thiruvananthapuram, India; ^3^ICAR- National Institute of Biotic Stress Management, Raipur, India; ^4^Division of Agricultural Extension, ICAR, KAB-1, New Delhi, India

**Keywords:** agroecological zones, cereal equivalent quantity, farmer FIRST program, interventions, food security

## Abstract

**Introduction:**

Nutrition-sensitive agricultural interventions are crucial in addressing malnutrition and promoting food security. The Farmer, Farm, Innovation, Resources, Science, and Technology (FIRST) Program is a national-level agricultural intervention program that was started in 2016 by the Indian Council of Agricultural Research (ICAR). Its primary objective is to transform the lives and livelihoods of Indian farmers, with a focus on income and livelihood security. This program envisages agricultural interventions ensuring national food security goals, with improved agricultural practices, enhanced food production, and increased access to nutritious food, especially for vulnerable populations.

**Methods:**

This study aims to investigate the food security improvement resulting from implementing nutrition-sensitive interventions introduced under the Farmer FIRST Program (FFP) in 15 agroecological zones. Four key indicators were employed to assess food security in the technology-focused field intervention: food availability, purchasing power, food gap, and food diversity. Food availability was measured at the macro level (state or national) using per capita food availability. However, at the micro level, particularly for farmers who produce their food and are secure in terms of availability, yield increases from the selected interventions under FFP served as an alternative measure. Purchasing power was assessed by the additional income generated to buy food during the off-season. The food gap was assessed using the cereal equivalent quantity (CEQ), which captures an aspect of consumers’ nutritional security. Its impact in India was evaluated using the propensity score matching technique with difference-in-difference (D-i-D) measure to estimate the unbiased overall effect on food security. Food diversity was captured using Barry’s index.

**Results:**

A sample of 2,282 respondents were interviewed from 2016 to 2020 to elicit data on the prevalence of undernutrition in India, which is 16.3%. The results revealed that post-intervention of the FFP, cereal availability increased by 147.74% in the Northern Plain, while pulses recorded over 200% growth in three regions. The incremental per capita farm income exceeded INR 20,000 in several zones, with the Northern Plain showing an increase of over 21 times. Food diversity improved marginally, supported by the integrated farming system. Overall, FFP interventions transformed nutritional security, benefiting 1,915 households, particularly in regions with historically low calorie intake, thereby demonstrating substantial gains in food security and living standards.

**Conclusion:**

The Farmer FIRST Program (FFP) has significantly improved dietary intake among participating households, enhancing food security. Nutrition-sensitive agricultural interventions under the FFP have reduced undernutrition by increasing food availability, boosting purchasing power, and narrowing the food gap.

## 1 Introduction

Since its independence, India has implemented several research-based and region-oriented development programs to attain food and nutritional security (1). As a result of these efforts, food grain productivity has increased substantially, leading to a surplus. With a steep increase in food grain production from 50 million tons during the 1950s to approximately 330.53 million tons in 2022–2023, India has attained the status of food exporter. As a result, the average dietary energy supply adequacy, a popular indicator of food security, for India in 2021 was 112%, indicating adequate food security (2). However, nutritional security has been a prime focus recently, mainly due to poor nutritional indicators (3). Over 2 billion people worldwide suffer from micronutrient deficiencies, indicating the widespread impact on global health and wellbeing (4–6). India accounts for the highest proportion of stunted children (31%), children with wasting (51%), under-five mortality (16%), and also has the most significant proportion of undernourished individuals in the world, according to 2018 data from the Food and Agriculture Organization (FAO) (7). Although the technology-led green revolution played a significant role in ensuring food security, the alarming level of malnutrition calls for a paradigm shift using technology-based strategies for achieving nutritional security. Emerging research paradigms (8) view food security as the function of food availability, accessibility, utilization, and asset creation. Technology-driven agricultural development has traditionally focused on maximizing productivity to make food available to the growing population. With the emergence of the Sustainable Development Goals (SDG) paradigm, food security is viewed as ensuring zero hunger by making nutrition-rich healthy food available to people experiencing poverty and people in need. This approach calls for “holistic” technology-based interventions by customizing the crop, horticultural, and animal systems to ensure continuous availability of nutritious foods to farm families and their neighborhoods. To achieve such conditions, the Indian Council of Agricultural Research (ICAR) implemented Farmer FIRST.^1^ Since 2016, the Farmer FIRST Programme (FFP) has focused on both food and nutrition security (9). In this program, technology and social inclusion act as key drivers of food security (10–13), with an added emphasis on ensuring nutritional security (14, 15).

The FFP envisages a path for attaining nutritional security, among other key SDG goals (16, 17), and follows a “technology assemblage approach” where 327 nutritionally rich crop-based packages, 252 horticultural packages, and 202 livestock packages were identified through a participatory approach and implemented in diverse agroecological zones (18). This program has been implemented since 2016–2017 at 52 centers through 11 regional-level Agricultural Technology Application Research Institutes (ATARI) as nodal organizations in 15 agroecological zones of India. Nutritional security is embedded in maximizing dietary diversity to attain nutritional security.

To achieve specific nutritional outcomes, agricultural interventions must address locally relevant food security challenges (19). The Farmer FIRST Program (FFP) provides location-specific agro-intervention services across 15 distinct agroecological zones, implementing agricultural technology packages designed to enhance livelihood, food, and nutritional security (20, 21). The FFP fosters stakeholder collaboration to tackle farming communities’ unique challenges (22, 23). Effective integration within the FFP is crucial for addressing these food and nutritional challenges (22, 24). Therefore, the program’s key objective is to promote innovation, stakeholder feedback, participation, diverse realities, varied methodological approaches, and targeted livelihood interventions to create an enabling environment for achieving food and nutritional security (25–28).

Defining and measuring nutritional security under the SDG framework is a challenging task. From a physiological perspective, nutritional security is attained when an individual maintains a nutritionally sufficient diet, and the biologically utilized food supports adequate growth, resilience, or recovery from illness, pregnancy, lactation, and physical exertion (29). However, an agricultural interventionist with an SDG perspective envisages nutritional security in terms of continuously maximizing the availability of nutritionally enriched food without days of hunger. The increased yield in the customized technology packages implemented through the FFP has ensured adequate food availability during harvest and later months. The additional income obtained through “technology packages” is utilized to buy food during the off-season. This strategy aims to address the non-availability of nutritious food during off-seasons and the limitations of low purchasing power, which past studies have identified as key “hindrances for food and nutritional security” (8, 30). The poor state of India’s nutritional security is due to external factors such as the pandemic, inflation, income losses, disruptions to the informal food market, and the lack of access to government safety net services (31). Individual factors such as reduced food access, loss of money, and consumption of an unbalanced diet were also observed among many farmers in the project implementation area (32–34).

Numerous indicators measure general food and nutritional security at global, national, household, and individual levels. Among the food security and nutritional security indicators, the FAO Indicator of Undernourishment (FAOIU) measures undernourishment as the percentage of the population whose dietary energy intake falls below the Minimum Dietary Energy Requirement. The Global Hunger Index (GHI) estimates hunger based on three dimensions (SDG-focused): insufficient food availability, nutritional shortfalls in children, and child mortality. The Global Food Security Index (GFSI) focuses on affordability, availability, and quality. The Diet Diversity Score (DDS) assesses nutritional adequacy, while medical and biomarker indicators (MBIs) are also widely used (35). These indicators reflect the prevalence of hunger and nutritional adequacy as food and nutritional security proxies. In agricultural technology interventions (36), targeted to maximize food availability and access at the household level, the food gap is a potential indicator of nutritional security (37). The food gap indicates the food required for the food-insecure population to achieve the specific caloric target (38). It measures the intensity of food insecurity at the aggregate level and is expressed as calories per capita per day or in grain-equivalent quantities. In the food gap approach, the cereal equivalent quantity (CEQ) is a standard nutritional assessment tool (39, 40).

Moreover, the D-i-D is the appropriate method for assessing the impact of interventions on household food security. It compares outcome changes over time between a treatment group (those receiving the intervention) and a control group (those not receiving the intervention). In addition to the food gap, factors such as food availability, income from interventions, and food diversity provide a fuller picture of food security comprehensively. Hence, this study aims to assess the impact of a nationwide technology intervention implemented through the FFP, with specific objectives including: (i) evaluating the impact of FFP interventions on food availability, purchasing power, and food diversity among farm households across 15 agroecological zones and (ii) assessing the reduction in the food gap and improvements in nutritional security resulting from the implementation of FFP interventions.

## 2 Materials and methods

### 2.1 Identification of study area

The FFP was implemented in 15 selected agroecological zones.^2^ From the country, a total sample of 2,433 farm households was selected proportionately from each FFP center and surveyed to assess food security. For each FFP center, 40 treatments and 10 controls were selected randomly for the survey. However, while testing and validating the data, only some data were deleted. Finally, 1,915 samples from the treatment group and 518 from the control group were retained for analysis (Table 1). The data were collected through a pre-tested interview schedule developed based on the food gap framework of assessing food security. The survey covering the period from September 2015 to August 2016 was administered in September 2016, while the survey for the period from March 2020 to February 2021 was conducted in March 2021. Moreover, the interventions under the FFP on field crops, horticultural crops, and livestock were chosen through participatory stakeholder analysis, involving scientists, farmers, and extension workers in collaboratively identifying field challenges and technological requirements. The study was conducted using the ethical guidelines for human experiments as laid down in the Declaration of Helsinki (41, 42). The studies involving human participants were reviewed and approved by the competent authority of our Academy, the National Academy of Agricultural Research Management (NAARM), Hyderabad. The participants provided their written informed consent to participate in this study.

**Table 1:** Sample selection—treatment and control.

S no	Agroecological zones	Zone no.	No. of FFP institutes	No. of treatment samples	No. of control samples	Total samples
1.	Western Plain, Kachchh, and part of Kathiwara Peninsula	2	3	120	31	151
2	Northern Plain and Central Highlands including Aravallis	4	14	563	143	706
3.	Central Malwa Highlands, Gujarat Plains, and Kathiawar Peninsula	5	2	80	20	100
4.	Deccan Plateau, hot semi-arid ecoregion	6	1	40	10	50
5.	Deccan (Telangana) Plateau and Eastern Ghats	7	3	103	32	135
6.	Eastern Ghats, Tamil Nadu Plateau, and Deccan (Karnataka)	8	3	110	26	136
7.	Northern Plain, hot sub-humid (dry) ecoregion	9	1	39	11	50
8.	Central Highlands (Malwa, Bundelkhand, and Eastern Satpura)	10	4	140	40	180
9.	Eastern Plateau (Chhattisgarh), hot sub-humid ecoregion	11	2	80	19	99
10.	Eastern (Chotanagpur) Plateau and Eastern Ghats	12	6	220	62	282
11.	Eastern Plain	13	2	79	37	116
12.	Western Himalayas	14	4	160	37	197
13.	North Eastern Hills (Purvanchal)	17	3	121	30	151
14.	Eastern Coastal Plain	18	1	40	10	50
15.	Western Ghats and Coastal Plain	19	1	20	10	30
Total				1,915	518	2,433

### 2.2 Evaluation strategy

The World Food Summit (1996) defines “Food Security” as “when all people, at all times, have physical and economic access to sufficient safe and nutritious food that meets their dietary needs and food preferences for an active and healthy life and it is measured in four dimensions, namely, Physical availability of food; Economic and physical access to food; and Food utilization; and stability” (43). In this study, the following measures were used to measure these four dimensions, namely, food availability, purchase power, food gap, and food diversity. However, since the intervention is a recent phenomenon, the stability aspect of food security is not explored in this study.

#### 2.2.1 Phase 1

##### 2.2.1.1 Food availability

Food availability is typically evaluated using the per capita availability of food at the macro level, such as at the state or national level. However, at the micro level, especially for the farmers who are food producers and food secure in terms of availability, the increase in yield resulting from selected intervention under the FFP is used in this study as a proxy for food availability.

##### 2.2.1.2 Purchase power

The purchasing power is evaluated as the incremental income generated through intervention, which can be used for buying food in the off-season. An increase in the household total income can be used as a proxy for food security as it has been associated with a 0.9% increase in the probability of households being food secure (44).

##### 2.2.1.3 Food diversity

The Herfindahl Index (HI), also known as the Herfindahl–Hirschman Index (HHI), is a measure of the concentration of items in a food basket (45). It is calculated by summing the squares of the shares of all food items in the basket. The formula is


$$HI=\overset{N}{\underset{i=1}{\sum }}{s_{i}}^{2}$$

where s_i_ is the share of food item i in a given space, and N is the number of items.

The Herfindahl Index (HI) ranges from 1/N to one.

A HI index below 0.01 (or 100) indicates high diversity.

A HI index below 0.1 (or 1,000) indicates moderate diversity.

A HI index between 0.1 to 0.18 (or 1,000–1,800) indicates moderate concentration.

A HI index above 0.18 (above 1,800) indicates high concentration.

Barry’s index (BI) is simply one minus Herfindahl’s index, and similarly, normalized Barry’s index (NBI) is one minus normalized Herfindahl’s index (46).


NBI=1–NH

##### 2.2.1.4 Food gap

The consumption of various food items, including cereals, pulses, milk, fish, eggs, meat, fruits, and vegetables, was compared between the treatment and control groups from 2016 to 2020 to evaluate the impact of the FFP on changing consumption patterns. Furthermore, the food gap was evaluated using the measure of cereal equivalent quantity (CEQ), which captures a dimension of the nutritional security of consumers (47). The CEQ is a multidimensional indicator encompassing various food groups, including cereals, pulses, milk, fish, eggs, fruits, vegetables, and other items. These were converted into cereal equivalents and aggregated into the CEQ using weights based on their relative nutritional contributions, following the method used by Rask and Rask (2011). Cereals are given a cereal equivalent (CE) conversion factor of 1. Other crops’ relative caloric contents of equal weights are equated to cereals. As an illustration, fruits have a lower concentration of calories per unit weight than grains, resulting in a CE factor value below one. The CEQ can be interpreted as cereal equivalent to the food in this study’s same input unit, kg/capita. The Food and Agriculture Organization Corporate Statistical Database (FAOSTAT) was used to collect information on the caloric content of crops.


$$CEQ=\overset{N}{\underset{i=1}{\sum }}w_{i}Q_{i}$$

where Qi = quantity consumed of “i”th item, w = weight of “i”th item. i = individual food groups, and N = number of food groups.

#### 2.2.2 Phase 2

**Figure 1:**
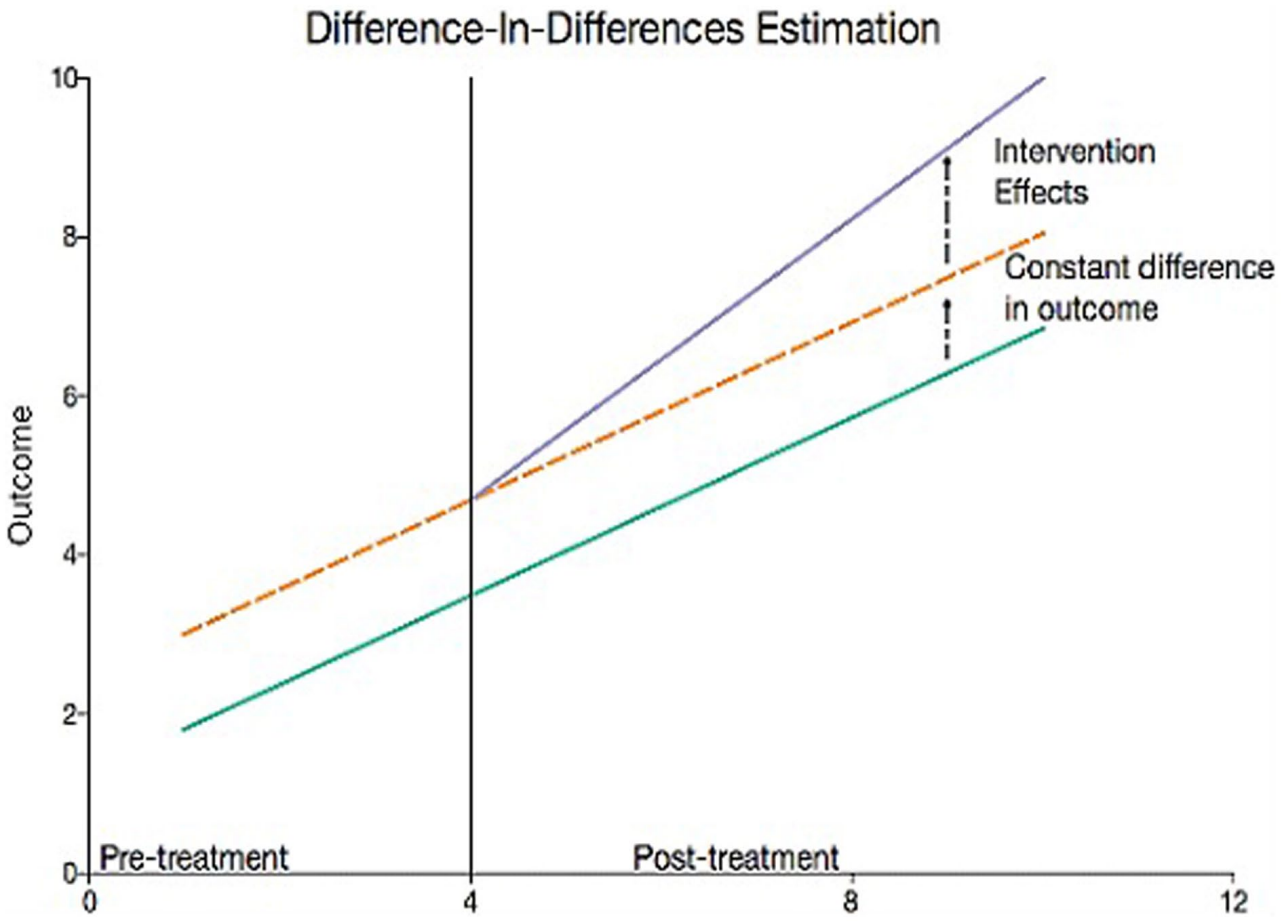
Graph for difference-in- difference estimation.

In the next phase, the impact of FFP interventions on nutritional security, i.e., the food gap, was assessed through the difference-in-difference method (D-i-D). In this method, the CEQ pertaining to the period 2019–2020 was calculated for treatment and control (no intervention) and compared to the base period 2016–2017 to assess the impact. Among the four indicators of nutritional security, the D-i-D was only used to measure the changes in food security concerning the food gap. The effect of other variables, such as food availability, food diversity, and purchase power, on food security was not subjected to the D-i-D due to drastic variations caused by the COVID-19 outbreak. Furthermore, the D-i-D variable was subjected to the propensity score matching (PSM) technique to weed out any issues related to the mismatch of treatment and control subjects. The D-i-D (Figure 1) was calculated using the following formula:


$$D-i-D_{i}=\Delta X_{\mathrm{iT}}–\Delta X_{iC}$$

where ΔX_i_ = ΔX_it_ – ΔX_i0_.

X_it_ = value of the ith item at current year “t” (year 2020), X_i0_ = value of the ith item at baseline year “0” (year 2016), T = treatment, and C = control.

i = 1 to 10 indicating different food groups, *viz.*, cereals, pulses, milk, egg, meat, fish, fruits, and vegetables.

The covariates used in the PSM model are age, educational status, and family size.

##### 2.2.2.1 Identification of pattern of FFP impact

To know whether the impact of the FFP has a pattern, the food gap estimated was superimposed on the nutritional data on household consumption during the period 2011–2012. National Sample Survey Organization (NSSO) conducts a large survey every 5 years wherein approximately 120,000 households are canvassed and information pertaining to the quantity and consumption of over 100 food items is captured. For this study, the latest available data, i.e., 68th Round pertaining to the year 2011–12, was used.

This study was conducted in regions with a high potential for adopting the FFP interventions. Consequently, the findings may not fully represent the impact across all areas within each agroecological zone. Further research focusing on specific intervention strategies in other agriculturally advanced regions is needed to better understand the broader applicability and impact of the FFP interventions.

## 3 Results and discussion

The main goal was to assess the influence of food security following the FFP interventions using selected variables. Four significant indicators, namely, food availability, purchase power, food gap, and food diversity, were evaluated to accomplish this.

### 3.1 Profile of selected farm and households

**Figure 2:**
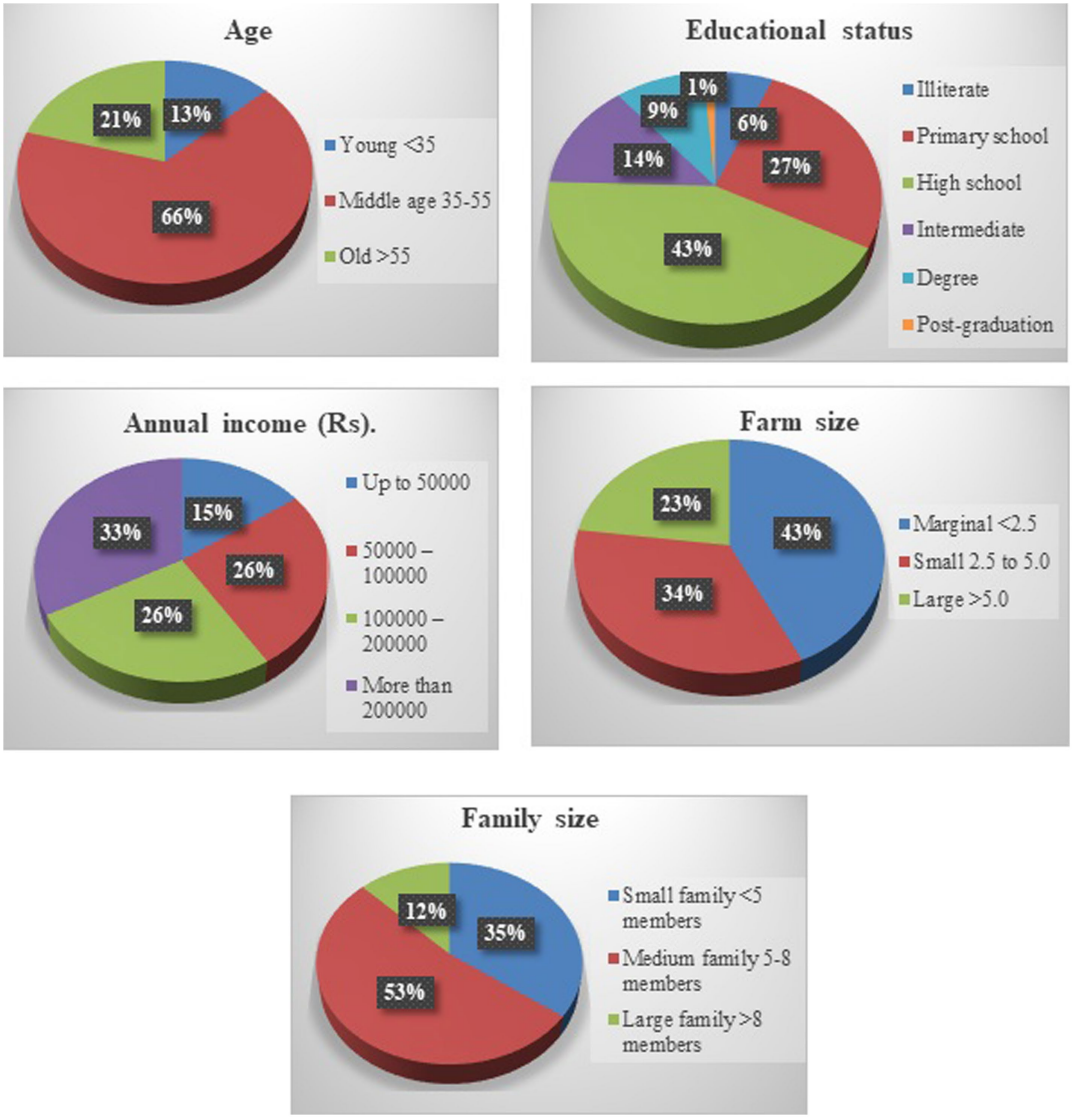
Profile characteristics of selected farm and households.

The profile of the respondents was delineated. The majority of the sample respondents were from the middle age group (66.32%), completed high school education (42.68%), earned over Indian Rupee (INR) 200,000 annually (32.89%), marginal farmers who own <1 ha of land (42.93%), and having 5 to 8 members in their family (52.63%) as shown in Figure 2.

### 3.2 Selection of interventions

The technology packages and interventions under the FFP were selected through a participatory stakeholder analysis process involving scientists, farmers, and extension workers in identifying field problems and technological needs. Table 2 shows the agroecological zone-wise details of these interventions of technology packages, such as field crops, horticultural crops, and livestock, tailored to those characteristics.

**Table 2:** Agroecological zone-wise agricultural and allied technology packages implemented in the FFP.

Zone no.	Agroecological zones	Crop based packages	Horticulture based packages	Livestock based packages	Total packages
Zone 2	Western Plain, Kachchh, and part of Kathiwara Peninsula	38 (45.24)	34 (40.48)	12 (14.28)	84
Zone 4	Northern Plain and Central Highlands including Aravallis	104 (48.60)	40 (18.69)	70 (32.71)	214
Zone 5	Central Malwa Highlands, Gujarat Plains, and Kathiawar Peninsula	7 (43.75)	6 (37.50)	3 (18.75)	16
Zone 6	Deccan Plateau, hot semi-arid ecoregion	4 (57.14)	1 (14.29)	2 (28.57)	7
Zone 7	Deccan (Telangana) Plateau and Eastern Ghats	19 (70.37)	4 (14.81)	4 (14.81)	27
Zone 8	Eastern Ghats, Tamil Nadu, Plateau, and Deccan (Karnataka)	13 (27.66)	16 (34.04)	18 (38.30)	47
Zone 9	Northern Plain, hot sub-humid (dry) ecoregion	8 (32.00)	9 (36.00)	8 (32.00)	25
Zone 10	Central Highlands (Malwa, Bundelkhand, and Eastern Satpura)	21 (19.09)	76 (69.09)	13 (11.82)	110
Zone 11	Eastern Plateau (Chhattisgarh), hot sub-humid ecoregion	21 (38.18)	20 (36.36)	14 (25.45)	55
Zone 12	Eastern (Chotanagpur) Plateau and Eastern Ghats	41 (48.81)	22 (26.19)	21 (25.00)	84
Zone 13	Eastern Plain	6 (35.29)	3 (17.65)	8 (47.06)	17
Zone 14	Western Himalayas	21 (48.84)	8 (18.60)	14 (32.56)	43
Zone 17	North Eastern Hills (Purvanchal)	8 (29.63)	9 (33.33)	10 (37.04)	27
Zone 18	Eastern Coastal Plain	11 (68.75)	3 (18.75)	2 (12.50)	16
Zone 19	Western Ghats and Coastal Plain	5 (55.56)	1 (11.11)	3 (33.33)	9

Values in parentheses indicate percentage.

As evident in Table 2, the number of interventions varies widely across the zones, from a minimum of 7 to 214. While the Northern Plain and Central Highlands, including the Aravallis, had 214 interventions, the Deccan Plateau, a hot semi-arid ecoregion, had only 7 interventions. Among the technology packages, 327 crop-based packages, 252 horticultural packages, and 202 livestock packages were implemented.

### 3.3 Change in food availability

The results showed that the FFP implementation has a positive impact on yield across all the zones, although the effect varies by agroecological zone and crop (Table 3). In the post-intervention phase, the food availability was recorded in the intervention regions. Regarding cereal-based technology interventions, the Northern Plain, a hot sub-humid (dry) ecoregion, produced the highest increase in food availability (147.74%). At the same time, Eastern Ghats, Tamil Nadu Plateau, and Deccan (Karnataka) have recorded the lowest (14.03%).

**Table 3:** Maximizing household food availability through increased crop yield from the FFP interventions (%).

Zone no.	Agroecological zones	Cereals	Oilseeds	Pulses	Vegetables and fruits
Zone 2	Western Plain, Kachchh, and part of Kathiwara Peninsula	28.83	33.33	233.33	111.71
Zone 4	Northern Plain and Central Highlands including Aravallis	39.61	42.35	33.33	4.00
Zone 5	Central Malwa Highlands, Gujarat Plains, and Kathiawar Peninsula	22.74	32.66	18.24	29.61
Zone 6	Deccan Plateau, hot semi-arid ecoregion	110.00	79.70	46.67	11.38
Zone 7	Deccan (Telangana) Plateau and Eastern Ghats	37.47	28.46	29.18	37.78
Zone 8	Eastern Ghats, Tamil Nadu Plateau, and Deccan (Karnataka)	14.03	50.89	34.12	45.21
Zone 9	Northern Plain, hot sub-humid (dry) ecoregion	147.74	62.02	257.32	20.00
Zone 10	Central Highlands (Malwa, Bundelkhand, and Eastern Satpura)	26.01	12.24	20.00	133.59
Zone 11	Eastern Plateau (Chhattisgarh), hot sub-humid ecoregion	28.26	13.74	35.14	23.53
Zone 12	Eastern (Chotanagpur) Plateau and Eastern Ghats	30.47	10.21	54.68	27.62
Zone 13	Eastern Plain	17.37	8.43	28.26	32.88
Zone 14	Western Himalayas	15.74	11.89	42.86	24.70
Zone 17	North Eastern Hills (Purvanchal)	98.72	–	26.50	25.00
Zone 18	Eastern Coastal Plain	87.21	46.32	266.06	50.09
Zone 19	Western Ghats and Coastal Plain	26.77	31.00	15.28	42.79

% change between treatment and control.

The highest food availability for the oilseeds was observed in the Deccan Plateau, a hot semi-arid ecoregion (79.70%), while Eastern Plains recorded the lowest oil seed availability (8.43%). The pulse intervention has produced a high impact in terms of an increase in pulse availability in three regions, namely, Eastern Coastal Plains, Northern Plain, hot sub-humid (dry) ecoregion, and Western Plain, Kachchh, and part of Kathiwara Peninsula, recording over 200% increase in pulse availability.

The fruit and vegetable interventions recorded over 100% increase in availability in two regions, namely, Central Highlands (Malwa, Bundelkhand, and Eastern Satpura) and Western Plain, Kachchh, and part of Kathiwara Peninsula. In contrast, the Northern Plain and Central Highlands, including the Aravallis region, had a meager 4% increase in availability. The yield has increased significantly due to promising interventions such as releasing high-yielding varieties and hybrids, better agronomic practices, and encouraging farmers to adopt advanced technologies through training and advisory systems provided through FFP.

### 3.4 Change in purchase power

The farm households’ purchase power change was assessed by the per capita farm income increase due to the FFP interventions. The results showed a significant increase in per capita farm income in all the zones where the FFP was implemented (Table 4). In many zones, the incremental per capita farm income was over INR 20,000, which translated to the tremendous purchasing power of the households. The increase in the incremental income ranged from 15.14% in the Eastern Ghats, Tamil Nadu Plateau, and Deccan (Karnataka) zone to 2130.17% in the Northern Plain and Central Highlands, including the Aravallis zone. Over 100% increase in the incremental income was observed in seven zones. The high increase in the incremental income in the Northern Plain and Central Highlands, including the Aravallis zone, is mainly due to a high marketable surplus of fruits and vegetables (96%) (Table 3). This indicates that the gain from FFP in some areas can not only address the food security of farm households but also transform their living standards.

**Table 4:** Change in per capita farm income due to the FFP interventions for the year 2020.

Zone no.	Agroecological zones	Per capita farm income (INR)		Incremental income (INR) (T-C)	% of increase
		Treatment	Control		
Zone 2	Western Plain, Kachchh, and part of Kathiwara Peninsula	59279.94	32858.22	26421.72	80.41
Zone 4	Northern Plain and Central Highlands including Aravallis	7264.10	325.72	6938.38	2130.17
Zone 5	Central Malwa Highlands, Gujarat Plains, and Kathiawar Peninsula	49349.88	26176.47	23173.41	88.53
Zone 6	Deccan Plateau, hot semi-arid ecoregion	55523.94	22755.56	32768.39	144.00
Zone 7	Deccan (Telangana) Plateau and Eastern Ghats	53160.73	29509.23	23651.50	80.15
Zone 8	Eastern Ghats, Tamil Nadu Plateau, and Deccan (Karnataka)	35540.17	30867.00	4673.17	15.14
Zone 9	Northern Plain, hot sub-humid (dry) ecoregion	27056.02	12396.40	14659.62	118.26
Zone 10	Central Highlands (Malwa, Bundelkhand, and Eastern Satpura)	42193.79	33604.53	8589.26	25.56
Zone 11	Eastern Plateau (Chhattisgarh), hot sub-humid ecoregion	42036.93	20836.70	21200.23	101.74
Zone 12	Eastern (Chotanagpur) Plateau and Eastern Ghats	20882.35	14350.88	6531.46	45.51
Zone 13	Eastern Plain	8786.17	4764.08	4022.09	84.43
Zone 14	Western Himalayas	13039.18	3333.42	9705.75	291.16
Zone 17	North Eastern Hills (Purvanchal)	25375.92	21805.36	3570.56	16.37
Zone 18	Eastern Coastal Plain	32957.32	10342.73	22614.59	218.65
Zone 19	Western Ghats and Coastal Plain	130556.2	19148.89	111407.40	581.80

### 3.5 Change in food diversity

The diversity of food intake, which reflects the food gap, indicates the food quality consumed by the farm households. The results showed that the food basket of the households in all the zones was diverse, and the FFP interventions had a marginal effect on diversity (Table 5). The changes in food diversity during the pre-and post-intervention period, as measured using the normalized Barry’s index, ranged from 0.00 in the Eastern Plain, the Eastern Ghats, Tamil Nadu Plateau, Deccan (Karnataka), and Central Highlands (Malwa, Bundelkhand, and Eastern Satpura) regions, to 0.14 in the Northern Plain, hot sub-humid (dry) ecoregion. However, it should be noted that the FFP has yet to lead to mono-cropping in any zone despite the release of better varieties and hybrids. One reason for holding this diversity in production is the implementation of the integrated farming system (IFS), a priority intervention module in the FFP. Several research studies have demonstrated that increasing food diversity has improved nutritional security. The Realigning Agriculture to Improve Nutrition (RAIN) program implemented in Zambia through customized agricultural interventions increased the crop diversity in the interventional area and food availability, thereby contributing to food security (48).

**Table 5:** Estimates of food diversity.

Zone no	Agroecological zones	Normalized Barry’s Index				Change in Barry’s index between 2016 and 2020
		2016		2020		
		Treatment	Control	Treatment	Control	
Zone 2	Western Plain, Kachchh, and part of Kathiwara Peninsula	0.67	0.66	0.76	0.73	0.10
Zone 4	Northern Plain and Central Highlands including Aravallis	0.71	0.71	0.72	0.72	0.01
Zone 5	Central Malwa Highlands, Gujarat Plains, and Kathiawar Peninsula	0.86	0.82	0.88	0.84	0.06
Zone 6	Deccan Plateau, hot semi-arid ecoregion	0.83	0.80	0.85	0.81	0.05
Zone 7	Deccan (Telangana) Plateau and Eastern Ghats	0.70	0.62	0.79	0.74	0.17
Zone 8	Eastern Ghats, Tamil Nadu Plateau, and Deccan (Karnataka)	0.83	0.84	0.84	0.84	0.00
Zone 9	Northern Plain, hot sub-humid (dry) ecoregion	0.70	0.71	0.85	0.85	0.14
Zone 10	Central Highlands (Malwa, Bundelkhand, and Eastern Satpura)	0.71	0.72	0.72	0.73	0.00
Zone 11	Eastern Plateau (Chhattisgarh), hot sub-humid ecoregion	0.40	0.40	0.84	0.78	0.44
Zone 12	Eastern (Chotanagpur) Plateau and Eastern Ghats	0.66	0.67	0.68	0.70	0.01
Zone 13	Eastern Plain	0.79	0.80	0.80	0.81	0.00
Zone 14	Western Himalayas	0.68	0.74	0.75	0.73	0.01
Zone 17	North Eastern Hills (Purvanchal)	0.90	0.88	0.84	0.84	0.02
Zone 18	Eastern Coastal Plain	0.81	0.74	0.84	0.74	0.10
Zone 19	Western Ghats and Coastal Plain	0.89	0.91	0.90	0.93	0.02

### 3.6 Evaluation of food gap

**Figure 3:**
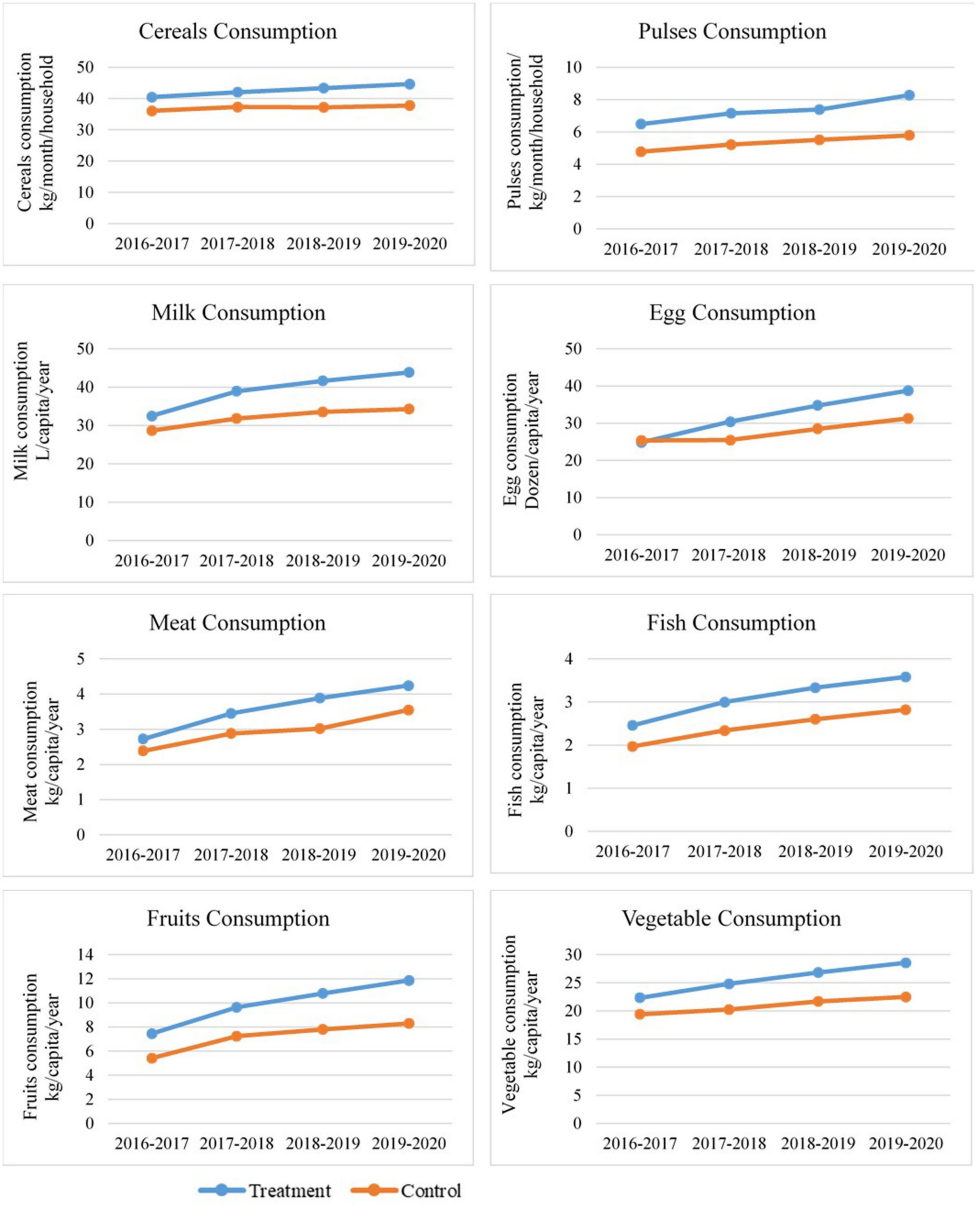
Consumption pattern of food among treated and control farmers from 2016–2017 to 2019–2020.

The FFP was implemented in 15 agroecological zones with interventions on field crops, horticultural crops, and livestock to enhance the nutritional security in those areas. There were changes in consumption patterns of cereals, pulses, milk, eggs, meat, fish, fruits, and vegetables after implementing the FFP. To assess these changes, these items were converted into cereal equivalents and aggregated into the CEQ, with weights assigned based on their relative nutritional contributions. The agroecological zone-wise cereal equivalent conversion factors for the food products are shown in Table 6. Then, the collected data were subjected to the D-i-D analysis to assess the significance of changes in the impact indicators in the treatment group over the control group. The estimates derived from the D-i-D estimator for the impact of the FFP interventions on nutritional security are shown in Table 7 and Figure 3. The changes in consumption due to the FFP intervention were assessed through a pre-post survey, which indicated changes in the impact indicators (Figure 3) for cereals, pulses, milk, eggs, meat, fish, fruits, and vegetables—key staple foods among farmers. The aforementioned per capita food consumption was previously relatively low among untreated farmers compared to treated farmers under the FFP.

**Table 6:** Cereal equivalent conversion factors for food products.

Zone no.	Agroecological zones	2016		2020	
		Control	Treatment	Control	Treatment
Zone 2	Western Plain, Kachchh, and part of Kathiwara Peninsula	46.00	40.86	48.88	48.10
Zone 4	Northern Plain and Central Highlands including Aravallis	43.67	46.82	49.07	55.38
Zone 5	Central Malwa Highlands, Gujarat Plains, and Kathiawar Peninsula	10.53	13.38	15.73	18.95
Zone 6	Deccan Plateau, hot semi-arid ecoregion	28.59	36.44	32.40	51.97
Zone 7	Deccan (Telangana) Plateau and Eastern Ghats	25.23	24.85	32.86	44.88
Zone 8	Eastern Ghats, Tamil Nadu Plateau, and Deccan (Karnataka)	52.07	51.99	52.63	52.63
Zone 9	Northern Plain, hot sub-humid (dry) ecoregion	36.27	39.04	77.39	86.20
Zone 10	Central Highlands (Malwa, Bundelkhand, and Eastern Satpura)	14.02	22.45	18.35	25.29
Zone 11	Eastern Plateau (Chhattisgarh), hot sub-humid ecoregion	12.87	13.16	18.42	29.00
Zone 12	Eastern (Chotanagpur) Plateau and Eastern Ghats	53.94	74.72	65.47	87.51
Zone 13	Eastern Plain	32.51	29.08	33.64	33.03
Zone 14	Western Himalayas	54.58	60.28	61.42	80.64
Zone 17	North Eastern Hills (Purvanchal)	31.28	26.93	20.41	19.14
Zone 18	Eastern Coastal Plain	20.58	42.40	21.14	67.43
Zone 19	Western Ghats and Coastal Plain	43.68	68.59	48.41	79.55

**Table 7:** Results of PSM technique on food security of different agroecological zones in India.

Zone no.	Agroecological zones	D-i-D of CEQ	P > (z)
Zone 2	Western Plain, Kachchh, and part of Kathiwara Peninsula	1.85	0.678
Zone 4	Northern Plain and Central Highlands including Aravallis	9.33*	0.021
Zone 5	Central Malwa Highlands, Gujarat Plains, and Kathiawar Peninsula	4.08*	0.011
Zone 6	Deccan Plateau, hot semi-arid ecoregion	13.88**	0.000
Zone 7	Deccan (Telangana) Plateau and Eastern Ghats	10.38*	0.027
Zone 8	Eastern Ghats, Tamil Nadu Plateau, and Deccan (Karnataka)	1.05	0.820
Zone 9	Northern Plain, hot sub-humid (dry) ecoregion	4.59	0.202
Zone 10	Central Highlands (Malwa, Bundelkhand, and Eastern Satpura)	8.7	0.831
Zone 11	Eastern Plateau (Chhattisgarh), hot sub-humid ecoregion	10.84**	0.002
Zone 12	Eastern (Chotanagpur) Plateau and Eastern Ghats	1.83	0.727
Zone 13	Eastern Plain	4.84	0.239
Zone 14	Western Himalayas	15.92	0.262
Zone 17	North Eastern Hills (Purvanchal)	5.67	0.240
Zone 18	Eastern Coastal Plain	4.18*	0.015
Zone 19	Western Ghats and Coastal Plain	4.18	0.239

* and ** denote significance at 5 and 1%, respectively.

**Figure 4:**
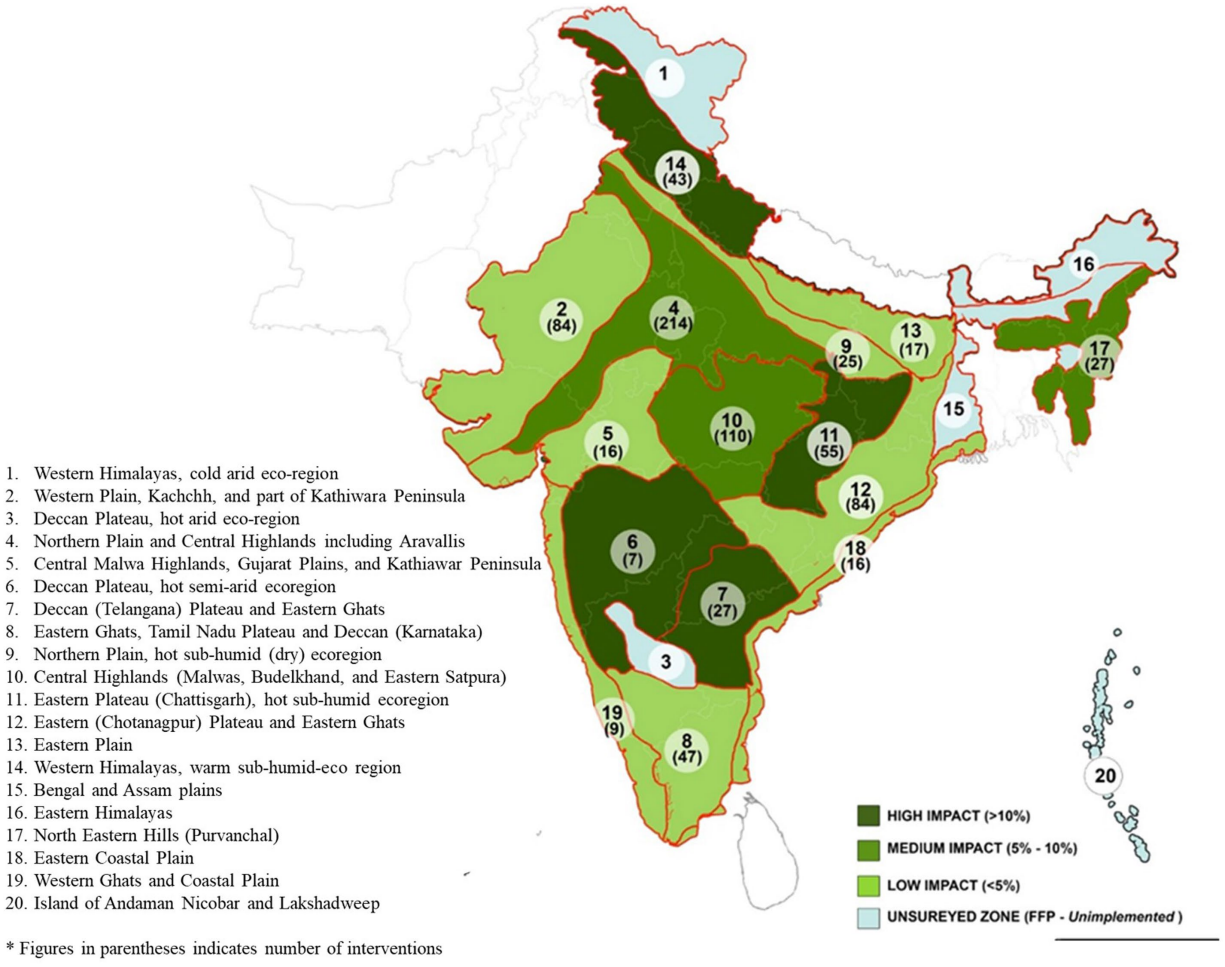
Impact on nutritional security in different agro ecological zones of India (NBSS and LUP) after FFP.

The execution of the FFP has created a remarkable transformation in food security since 2016. Significant reduction in the food gap due to FFP interventions was observed in Northern Plain and Central Highlands, including the Aravallis, Central Malwa Highlands, Gujarat Plains, Kathiawar Peninsula, Deccan Plateau, hot semi-arid ecoregion, Eastern Plateau (Chhattisgarh), hot sub-humid ecoregion, and Eastern Coastal Plains (*p* < 0.05). Figure 4 illustrates the geographical area covering high impact, medium impact, and low impact of the FFP farmer’s status of food security in different agroecological zones, and the above heatmap shows the D-i-D of the CEQ status of food security in different agroecological zones of India ranging from highest to lowest in light green to dark green color. An agricultural intervention study conducted in Ethiopia (49) has indicated that increased access to food obtained through agricultural interventions has significantly reduced the food gap. The increasing importance of markets in understanding how agriculture impacts human nutrition (50), alongside the environmental and climatic context (51), are two of the most critical factors influencing the connections between agriculture and nutrition. The treated farmers experienced a moderate rise in food consumption compared to the control farmers, who experienced a minor increase over the years. The farmers could implement location-specific agricultural interventions, allowing them to boost income and invest in their consumption patterns. Over the years, this program has done its best to turn undernourished people into nourished people. In 1915, the number of households improved through nutritional level and composition through interventions under the FFP. Among the 15 agroecological zones, Eastern Plain, followed by Eastern Coastal Plain and Central Malwa Highlands, Gujarat Plains, and Kathiawar Peninsula, observed the highest D-i-D, and the least was observed in Eastern Ghats, Tamil Nadu Plateau, and Deccan (Karnataka).

The FFP interventions significantly improved food security indicators, including availability, purchasing power, and food diversity across the agroecological zones. Households experienced notable per capita farm income increases, with some zones reporting increments of over INR 20,000, enhancing their purchasing power. Implementing these interventions led to substantial improvements in food availability, particularly in pulses and cereals, with increases exceeding 200% in some regions. While overall food diversity showed only marginal improvements, the integrated farming system (IFS) effectively maintained a diverse production landscape. Ultimately, the FFP transformed the nutritional status of 1,915 households, demonstrating its positive impact on food security in the targeted areas.

**Figure 5:**
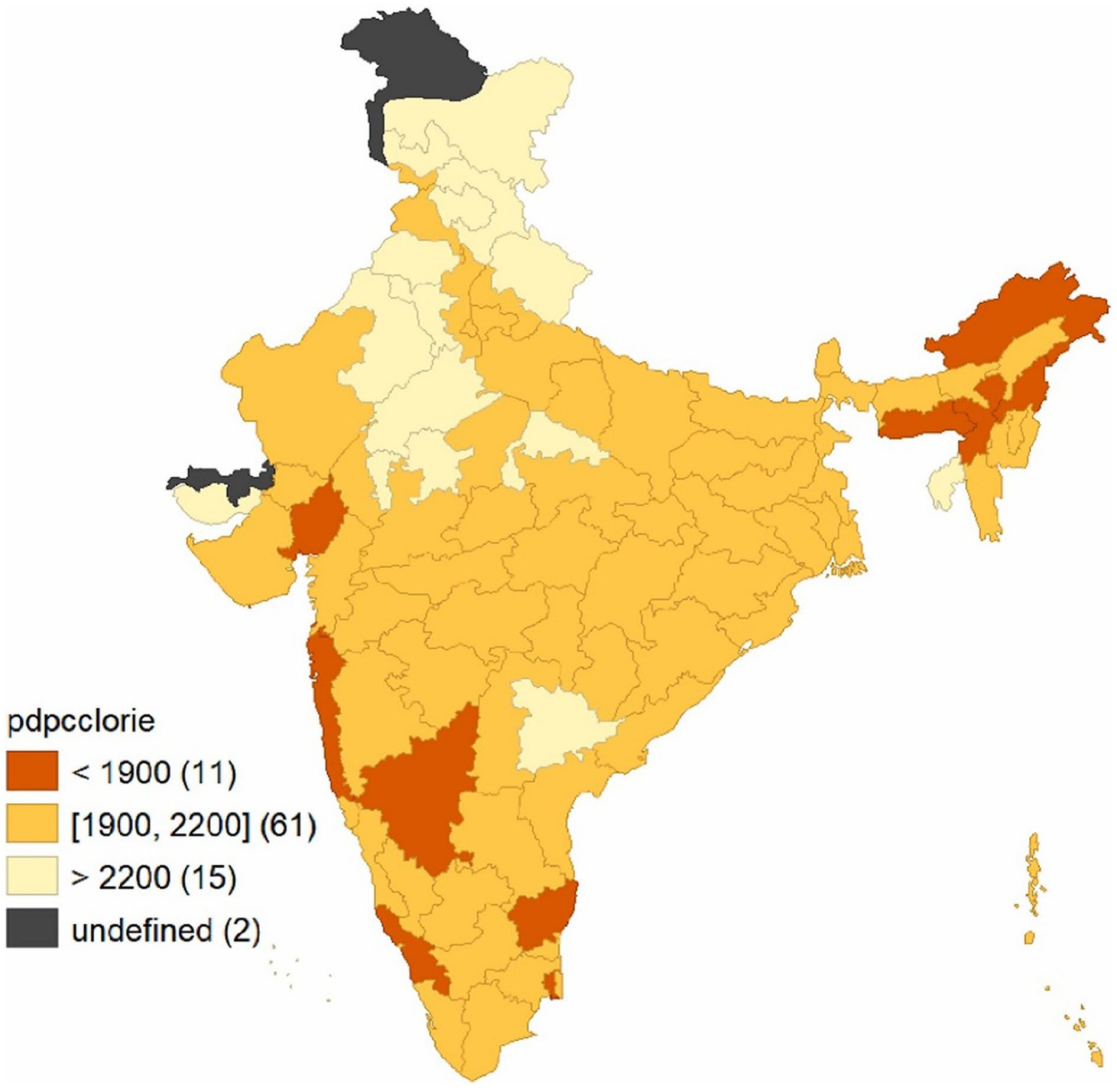
Regional pattern of calories (per day per capita Kcal) intake across the regions of India, NSS, 2011–12.

Furthermore, the impact of FFP was compared to the nutritional levels during 2011–2012 to see whether the impact was exhibiting any pattern. The data on the consumption of Indian households during 2011–2012 were elicited from unit-level data of the National Sample Survey and used in this study for comparison. The regional pattern of calorie intake in India pertaining to the nutritional status of households is shown in Figure 5. The map highlights the variation in per day per capita calorie consumption across different regions in India. Accordingly, there are approximately 11 regions in India where the per day per capita calorie consumption is <1900 Kcal. It can be seen that, by superimposing with an impact map (Figure 4), the FFP was found to have a moderate-to-high level of impact in these regions. These regions are mainly clustered in North Eastern India (52), parts of Maharashtra, Kerala, Karnataka, and Tamil Nadu. On the other hand, in areas with more than 2,200 Kcal of per day per capita calorie intake (15 regions), the impact of the FFP was mostly moderate. This reveals that the impact of the FFP has a pattern and the impact is more pronounced in areas where malnutrition is high and moderate in other regions.

This study has provided enough evidence for the impact of the FFP interventions on food security by assessing its various dimensions in terms of access, availability, stability, utilization, and agency (53).

## 4 Conclusion

Ensuring food security has emerged as a critical national agenda, notably as India ranks low on nutritional indicators and is home to the world’s most significant proportion of undernourished people. Agricultural development, supported by technological interventions, plays a vital role in addressing the country’s food and nutritional security. The FFP has significantly impacted food security across 15 agroecological zones in India, contributing to many SDGs, including nutritional security. The participatory design of the interventions under the FFP ensured their contextual appropriateness, with a total of 781 tailored packages addressing specific regional challenges. Enhanced purchasing power, with income gains exceeding INR 20,000 in many zones, underscores the program’s role in boosting household economic resilience. The D-i-D measure using the propensity score matching technique confirmed that the FFP significantly enhanced dietary diversity and overall nutritional security among participating farm households. With its holistic approach, encompassing field crops, horticulture, and livestock interventions, the program effectively addressed regional food insecurity, achieving notable improvements in areas such as the Eastern Plains and Central Malwa Highlands. By implementing targeted agricultural interventions, the FFP has improved vital food security indicators, such as food availability, purchasing power, and food gap, resulting in increased yields, higher household incomes, and improved nutritional intake. Significant reductions in food gaps and increased per capita cereal-equivalent consumption demonstrate the program’s success in improving nutritional security, particularly in regions with lower baseline calorie consumption. Overall, the FFP interventions have profoundly transformed food security and nutritional status for 1,915 households, offering a scalable model for addressing malnutrition and promoting sustainable agricultural development in resource-constrained regions.

## 5 Policy implication

This study proposes the following policy implications and strategies for upscaling and outscaling the FFP.

- *Shift agricultural policies toward low-productivity regions:* To ensure food security, agricultural pricing and policy frameworks must prioritize harnessing the untapped potential of low-productivity regions.
- *Adopt a farmer-centric approach:* Policies must actively place farmers at the core of decision-making in food security governance. This includes involving them in problem identification, prioritization, experimentation, and management, which is the core of the FFP.
- *Strengthen farmer–scientist collaboration:* Programs such as the FFP demonstrate the importance of fostering collaboration between farmers and scientists to develop, apply, and adapt technologies that address real-world challenges.
- *Leverage high-income growth potential:* Specific interventions under the FFP, such as promoting high-value crops and improved practices, have shown exceptional potential for increasing farm incomes. Scaling these interventions in targeted regions can serve as a model for boosting economic and food security.
- *Promote crop diversification:* The marginal improvements in food diversity underscore the need for policies encouraging diverse cropping systems. This can be achieved through incentive mechanisms for integrated farming systems and support for cultivating nutrient-rich and high-value crops.
- *Integrate local and global policy goals:* Aligning national agricultural initiatives such as the FFP with global nutrition and food security targets ensures coherence in tackling food insecurity. This includes leveraging methodologies such as propensity score matching (PSM) to design evidence-based interventions adaptable worldwide.
- *Replicate FFP in similar ecological contexts:* The FFP’s success in integrating innovation, feedback mechanisms, and multi-stakeholder participation makes it a replicable model for addressing food security challenges in other countries with slow agricultural technology adoption.

## Data Availability

The raw data supporting the conclusions of this article will be made available by the authors, without undue reservation.

## Footnotes

^1^Farm Innovation Resources Science and Technology.

^2^Agroecological regions by the National Bureau of Soil Survey and Land Use Planning (NBSS and LUP).
